# Sp17 Protein Expression and Major Histocompatibility Class I and II Epitope Presentation in Diffuse Large B Cell Lymphoma Patients

**DOI:** 10.1155/2017/6527306

**Published:** 2017-10-24

**Authors:** Kamel Ait-Tahar, Amanda P. Anderson, Martin Barnardo, Graham P. Collins, Chris S. R. Hatton, Alison H. Banham, Karen Pulford

**Affiliations:** ^1^Nuffield Division of Clinical Laboratory Sciences, Radcliffe Department of Medicine, University of Oxford, Oxford, UK; ^2^Transplant Immunology & Immunogenetics, Oxford Transplant Centre, Churchill Hospital, Oxford, UK; ^3^Department of Clinical Haematology, Churchill Hospital, Oxford, UK

## Abstract

Improved therapies are urgently needed for patients with diffuse large B cell lymphoma (DLBCL). Success using immune checkpoint inhibitors and chimeric antigen receptor T cell technology has fuelled demand for validated cancer epitopes. Immunogenic cancer testis antigens (CTAs), with their widespread expression in many tumours but highly restricted normal tissue distribution, represent attractive immunotherapeutic targets that may improve treatment options for DLBCL and other malignancies. Sperm protein 17 (Sp17), a CTA reported to be immunogenic in ovarian cancer and myeloma patients, is expressed in DLBCL. The aim of the present study was to investigate Sp17 epitope presentation via the presence of a cytotoxic T cell (CTL) and a CD4 T-helper (Th) response in DLBCL patients. A significant *γ*-interferon CTL response was detected in peripheral blood mononuclear cells of 13/31 DLBCL patients following short-term cell stimulation with two novel HLA-A^⁎^0201 peptides and one previously reported HLA-A^⁎^0101-restricted nine-mer Sp17 peptide. No significant responses were detected in the HLA-A^⁎^0201-negative DLBCL patients or four healthy subjects. A novel immunogenic 20-mer CD4 Th Sp17 peptide was detected in 8/17 DLBCL patients. This is the first report of a CTL and a CD4 Th response to Sp17 in DLBCL and supports Sp17 as a potential immunotherapeutic target for DLBCL.

## 1. Introduction

DLBCL is the most common form of mature B cell lymphoma and is heterogeneous with respect to morphology, clinical features, and immunophenotype [1]. A significant proportion of patients with DLBCL fail to achieve long-term remission despite advances in the definition of clinically relevant subtypes and treatment [2]. The development of improved immunotherapeutic options for the treatment of DLBCL thus remains urgent. The recent breakthroughs using immune checkpoint inhibitors and chimeric antigen receptor (CAR) T cell technology have opened up new avenues for achieving these objectives [3, 4]. However, the identification of relevant antigenic targets, particularly those presented on the cell surface of primary tumours and not just cancer cell lines, remains a priority.

Tumour-associated antigens (TAAs), recognized by the immune system of the patient, have been studied extensively in haematological malignancies. Evidence in support of their potential therapeutic benefit has been provided by autologous bone marrow transplantation and donor lymphocyte infusion studies, demonstrating that donor cells can recognize and respond to TAAs in a variety of malignancies, such as multiple myeloma and myeloid leukaemia [5, 6]. TAAs that are of current particular interest for improving treatment regimens are the family of cancer testis antigens (CTAs). Previous studies have reported expression of various CTAs in haematological malignancies, such as lymphomas [7, 8] and myeloid malignancies [9, 10], and in multiple myeloma [11–15]. These immunogenic molecules are highly tumour-specific and frequently expressed in various types of cancer, properties which make them promising candidate targets for cancer immunotherapy, including cancer vaccination and adoptive T cell transfer with chimeric T cell receptors [16–18].

One of the CTAs under intense investigation is the Sp17 protein. Its restricted expression in testis and its reported expression and immunogenicity in ovarian cancer [19–21], non-small cell lung cancer [22], and myeloma [23–25] patients make it an attractive candidate immunotherapeutic target in these malignancies. Moreover, Sp17 was found to be present on the surface of malignant lymphoid cells, including B- and T-lymphoid cell lines, and on the surface of primary cells isolated from two patients having B-lymphoid tumours [26]. We have also previously reported Sp17 protein expression in both primary DLBCL and DLBCL cell lines [27]. It has been suggested that Sp17 is an “oncofetal antigen” since it is expressed in embryonic as well as adult neoplastic cells, but not in normal tissues, and has been associated with the motility and migratory capacity of tumour cells [28]. As demonstrated with other CTAs, this functional role in tumour biology makes Sp17 a good immunotherapeutic target as it is unlikely to lose expression under therapeutic selection pressure. The current study was performed to explore the immunogenicity of the Sp17 protein and its expression in DLBCL patients.

## 2. Materials and Methods

### 2.1. Subjects and Samples

Peripheral blood was obtained from 31 patients with B cell lymphoma attending the Haematology Departments of the John Radcliffe Hospital, Oxford (*n* = 25), and Milton Keynes General Hospital (*n* = 6). The patient cohort (previously described in earlier studies on the PASD1 CTA [29, 30]) consisted of 22 patients with de novo DLBCL (two with relapsed DLBCL), seven patients with transformed DLBCL, and two patients with T cell rich B cell lymphoma. The patients presented with differing stages of disease and their clinical details and treatment protocols are summarized in Supplementary Table  1 in Supplementary Material available online at https://doi.org/10.1155/2017/6527306. Normal testis and tonsil tissues were obtained from the Department of Pathology, John Radcliffe Hospital, and used as positive and negative controls, respectively. Peripheral blood samples were also obtained from four healthy subjects. HLA typing was done by polymerase chain reaction (PCR) as previously described [31]. Ethical approval and written consent were obtained from the Oxfordshire Research Ethics Committee B (C02.356) for all blood samples collected and tissue sections used in the immunolabelling studies.

### 2.2. Peptides

CTL peptides: Two 9-amino-acid peptides, predicted with high binding affinity to the major histocompatibility complex (MHC) class I HLA-A^*∗*^0201 allele, were identified using the web-based SYPETHI (http://www.syfpeithi.de) and the HLA peptide prediction site of the Bioinformatics and Molecular Analysis Section (BIMAS, National Institutes of Health, Bethesda, USA) programmes. The peptides identified were as follows: Sp17(1)_43–52_ (SLLEKREKT); Sp17(2)_19–27_ (LLEGLTREI). Sp17(3)_102–110_ (ILDSSEEDK) previously identified as immunogenic in an HLA-A1 healthy donor [24] was also investigated. A control irrelevant peptide from the HIV-1 reverse transcriptase (ILKEPVHGV) that binds to HLA-A^*∗*^0201 was used in the CD8 T cell ELISPOT assays (Invitrogen, Paisley, UK). The Sp17 peptides were synthesized by standard chemistry on a multiple peptide synthesizer (Proimmune, Oxford, UK) and were >90% pure. Lyophilized peptides were dissolved in dimethyl sulfoxide and stored at −20°C.

*CD4 T-Helper (Th) Peptides*. The TEPITOPE prediction algorithm and SYPETHI (http://www.syfpeithi.de) programs were used to select three 20-mer Sp17 peptides predicted to be immunogenic in the context of HLA-DRB1 ^*∗*^0101, ^*∗*^0301, ^*∗*^0401, ^*∗*^0701, ^*∗*^1101, and ^*∗*^1501 (the most prevalent alleles among the Caucasian population) [31]. The peptides identified were as follows: Sp17(4)_9–28_ (YRIPQGFGNLLEGLTREILR); Sp17(5)_67–86_ (FYNNHAFEEQEPPEKSDPKQ); and Sp17(6)_118–139_ (VKIQAAFRGHIAREEAKKMK). The irrelevant control peptide was HIV-1_121–140_ (DESFRKYTAFTIPSMNNETP) (Invitrogen, Paisley, UK).

### 2.3. Antibodies

The anti-Sp17 monoclonal antibody was a kind gift from S. H. Lim (Texas). Antibodies to BCL6 (PG-B6p), CD10 (56C6), CD4 (T4-10, IgG1 isotype), and CD20 (DAKO-L26, IgG2a isotype) were purchased from DakoCytomation (Ely, Cambridgeshire, UK) while anti-CD8 (X-107, IgG1 isotype, used undiluted) was prepared in the authors' laboratory. Anti-MUM1 was a kind gift from Professor B. Fallini (Perugia, Italy). The anti-HLA-A^*∗*^0201 (BB7.2) used in the CD8 T cell blocking experiments was purchased from BD BioSciences (Oxford, UK). Major histocompatibility complex (MHC) class II expression was studied using the monoclonal anti-HLA-DP, DQ, and DR antibody (CR3/43) (DakoCytomation, Glostrup, Denmark). Unless stated, all antibodies were used at dilutions recommended by the manufacturers. Rabbit anti-CD3 (DAKO-CD3, diluted 1 : 100) and the Envision-horseradish peroxidase (HRP) labelling system were obtained from DakoCytomation. The MACH 3™ HRP-polymer detection kit was purchased from Biocare Medical (Wokingham, Berkshire, UK). Goat anti-rabbit immunoglobulin (Ig) and anti-mouse Ig-isotype specific antibodies conjugated to either fluorescein isothiocyanate (FITC) or Texas Red™ (diluted 1 : 100) were obtained from Invitrogen Ltd. (Paisley, UK).

### 2.4. Immunolabelling

Paraffin-embedded tissue sections were dewaxed and heat-induced antigen retrieval was performed using 50 mM Tris: 2 mM EDTA at pH 9.0. Immunolabelling for anti-MHC-class II was carried out using the Envision-HRP labelling kit (DAKO, Ely, UK). The method of staining for Sp17 protein expression and the subtyping of the DLBCL cases (germinal or nongerminal center subtypes [32]) was performed as previously described [27].

### 2.5. Preparation and Culture of PBMCs

Peripheral blood mononuclear cells (PBMCs) were prepared in RPMI 1640 medium containing 10% fetal calf serum (FCS) (RPMI 1640/FCS, Invitrogen Ltd.) as described previously [33]. PBMCs (0.5 × 10^5^) in 200 *μ*l of RPMI 1640/FCS were added to each well of a 96-well round-bottomed plate and incubated for 8–10 days with 10 *μ*mol of one of the following: Sp17(1), Sp17(2), Sp17(3), Sp17(4), Sp17(5), Sp17(6), or the control HIV peptides, 10 *μ*g/ml phytohaemagglutinin (PHA; Sigma-Aldrich Co. Ltd., Dorset, UK), or tissue culture media only. Recombinant interleukin-2 (rIL-2: 20 iu/ml; Roche Diagnostics, Indianapolis, IN, USA) and rIL-7 (25 ng/ml; R&D Systems, Minneapolis, MN, USA) were added on days 2, 5, and 7.

### 2.6. Enzyme-Linked Immunospot Assay (ELISPOT)

After 8–10 days of culture, the cells were washed and incubated for 18 h with RPMI 1640/FCS at 37°C in 5% CO_2_ with one of the Sp17 peptides, HIV control peptide, PHA, or medium only. Peptides were used at 10 *μ*mol and all cultures were carried out in triplicate. Gamma-interferon (*γ*-IFN) release assays were performed according to manufacturer's instructions (Mabtech, Stockholm, Sweden). Spots were counted using an automated ELISPOT reader (Autoimmun-Diagnostika, Strasberg, Germany). Results were considered positive if the number of spots in the test wells was at least twice those present in the control cultures (media only or containing the irrelevant HIV-1 peptide) and assays were excluded if there were more than 25 spots per well in the absence of peptides. All tests were performed in triplicate.

### 2.7. Generation of CD8 T Cell Lines, Depletion, and Blocking Experiments

PBMCs cultured at a density of 2 × 10^6^ cells/ml were cultured in RPMI 1640/FCS containing 10 *μ*mol of the appropriate Sp17 peptides. After 72 h, an equal volume of RPMI 1640/FCS containing 50 IU of rIL-2 per ml was added. Half of the medium was removed and replaced with fresh medium every 3 d. The cells were restimulated weekly for 6 weeks with the Sp17 peptides before being used in an ELISPOT assay. In some experiments, CD8-positive T cells were enriched from the CTL lines using magnetic beads coated with anti-human CD8 antibody according to the manufacturer's instructions (Dynabeads, Dynal, Oslo, Norway) before assay. In other experiments, the anti-HLA-A^*∗*^0201 antibody (BB7.2) was added at a concentration of 10 *μ*g/ml to block *γ*-IFN release.

### 2.8. Statistical Analysis

Student's *t*-test was used to analyse the results obtained in the ELISPOT assays and the immune response while Fisher's exact test was used to analyse the presence of a CTL response with Sp17 protein expression in patients who were HLA-A^*∗*^0201 or HLA-A^*∗*^0101 positive. *P* values < 0.05 were considered significant.

## 3. Results

### 3.1. Sp17 Protein Expression in DLBCL Patient Biopsies

Routinely fixed tissue sections from diagnostic biopsies were available for 20 of the 31 DLBCL patients to investigate Sp17 protein expression by immunohistochemistry (IHC). Clinicopathological characteristics of the DLBCL patients including their cell-of-origin classification, results of the Sp17 immunolabelling of tumour biopsies, and MHC class I and II expression are summarized in Tables 1 and 2 and Supplementary Table  1. Labelling with the Sp17 antibody was detected in the tumour cells derived from nine patients. Both nuclear and cytoplasmic labelling of Sp17 were observed in five cases, while scattered nuclear labelling was observed in the remaining four cases. Sp17 protein was not detected in the remaining 11 patients. In normal testis sections, the anti-Sp17 antibody detected weak staining of protein in the cytoplasm of the spermatogonia and in the cytoplasm and nuclei of the primary spermatocytes and the spermatozoa (strong staining). Sp17 protein expression was absent in normal tonsil. These data are consistent with those illustrated in our initial pilot study of Sp17 expression in DLBCL [27].

### 3.2. CD8 T Cell Responses to Sp17

The results of the *γ*-IFN response ELISPOT assay are summarized in Table 1. Significant *γ*-IFN responses to Sp17 were observed in 14/31 DLBCL patients after short-term culture with the Sp17 peptides compared to those results obtained from the control cultures (cells stimulated with the irrelevant HIV peptide or medium only, *P* < 0.05) and eight patients showed a response to more than one peptide. The Sp17 *γ*-IFN responses correlated with Sp17 protein expression in eight patients expressing Sp17 (0.0497, *P* < 0.05). With the exception of patients (9 and 39) who had a response to Sp17(1) peptide, no significant *γ*-IFN responses were detected in the eight patients with tumours where Sp17 protein was not detected. This suggests that the responses are antigen-driven. Two of the five HLA-A^*∗*^0101-positive patients (7 and 39) responded to the Sp17(3) peptide, previously identified as immunogenic in an HLA-A1 healthy donor [24]. Conversely, none of the HLA-A^*∗*^0201-positive patients responded to the Sp17(3) peptide, possibly reflecting the HLA-restricted nature of the responses. Frequencies of Sp17-responding T cells varied between patients, ranging from 1 : 600 (0.2%) PBMCs in patient 8 to 1 : 2000 (0.05%) in patient 39. Sp17(1) was identified as the most immunogenic peptide since it induced significant responses in the majority of the responding patients (13/15). No significant responses to any of the Sp17 peptides were detected in the four healthy donors examined.

### 3.3. CD4 T Cell Responses to Sp17

CD4 Th responses were examined in PBMCs of 17 DLBCL patients, eight of which displayed Sp17 protein expression. A significant response to Sp17(4) was observed in 8/17 patients (Table 2). None of the patients displayed a significant response to either Sp17(5) or Sp17(6). Patients 8 and 12 displayed the highest CD4 Th responses. It is noteworthy that some of the patients who responded most vigorously to either of the CTL peptides Sp17(1) and Sp17(2) (Table 1) also displayed the highest responses to the Sp17(4) peptide (patients 1, 8, and 12 in Table 2). Seven patients with a CD4 Th response also exhibited a CTL response to Sp17.

### 3.4. Persistence and Specificity of the *γ*-IFN Response to Sp17

**Figure 1:**
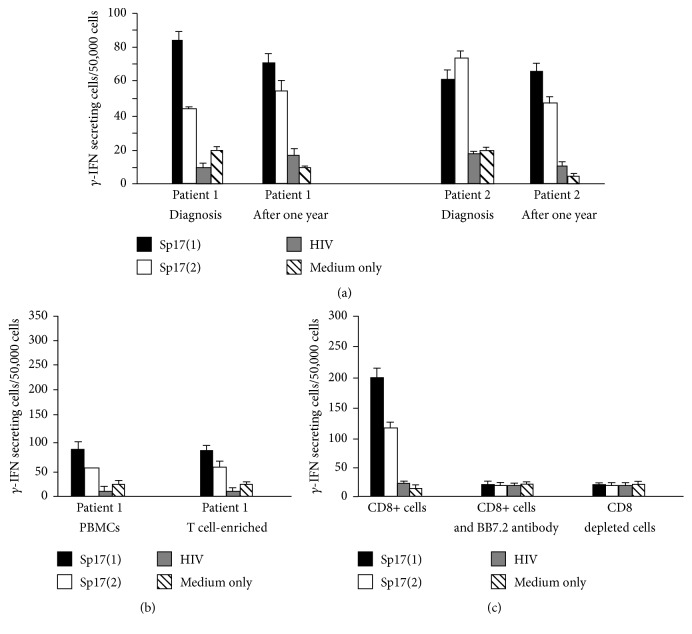
*γ*-IFN responses of patients 1 and 2 to Sp17 peptides. In (a) peripheral blood mononuclear cells (PBMCs) obtained from patients 1 and 2 at time of diagnosis after one year from start of treatment were maintained in short tern culture. A significant *γ*-IFN response to peptides Sp17(1) and Sp17(2) was observed in cells from both patients obtained at both time points (*P* < 0.05). No significant response was detected in cultures stimulated by the HIV peptide or containing medium only. (b) PBMCs from patient 1 after three rounds of peptide stimulation expanded in response to Sp17(1) and Sp17(2) peptides. (c) PBMCs from patient 1 were either enriched for CD8-positive cells using anti-CD8 antibody-coated magnetic beads or incubated with an anti-HLA-A^*∗*^0201 monoclonal antibody (BB7.2). A significant *γ*-IFN response was observed only in the culture containing the CD^*∗*^-positive cells in the absence of anti-MHC class I (*P* < 0.05). No significant responses were detected in the control cultures or the irrelevant peptides. The results are mean +/− SD and were obtained from triplicate ELISPOT cultures.

The CD8 T cell responses to Sp17(1) and Sp17(2) were investigated in samples from two HLA-A^*∗*^0201-positive patients (1 and 2), who were both in remission one year after initial diagnosis. A significant *γ*-IFN response to both Sp17 peptides was still detectable in the two patients after one year (Figure 1(a)) suggesting the persistence of a pool of circulating memory CD8-positive T cells to the Sp17 protein.

PBMCs from the HLA-A^*∗*^0201-positive patient 1 were maintained in culture to permit further analysis of their functional activity. PBMCs were restimulated weekly with rIL-2 and with one of the following: Sp17(1), Sp17(2), or the irrelevant HIV peptide. After 3 weeks, cells were tested for their *γ*-IFN secreting activity to the Sp17 and control peptides in an overnight ELISPOT assay. T cells were found to expand and respond specifically to both Sp17 peptides. After three rounds of expansion, Sp17(1)- and Sp17(2)-specific CD8+ T cells increased almost three- and twofold, respectively, compared to the nonexpanded population (Figure 1(b)). The CD8-enriched T cell *γ*-IFN response to the Sp17 peptides was abrogated by the removal of CD8-positive T cells or by the addition of the anti-HLA-A^*∗*^0201 monoclonal antibody BB7.2 (Figure 1(c)). These results confirm the CD8-positive, MHC class I restricted nature of the response.

### 3.5. CTL and CD4 Th Responses to Both the Sp17 and PASD1 CTAs

Results of the Sp17 T cell responses reported here were compared to those obtained for the PASD1 antigen in two previous studies using cells from the same cohort of DLBCL patients [29, 30]. Cells from ten patients were able to mount CTL responses to both Sp17 and PASD1 CTAs (Supplementary Table  2). CD4 Th responses to both the PASD1 and Sp17 antigens were also detected in five of these patients.

## 4. Discussion

The Sp17 protein is a member of the CT-X group of CTAs, those whose members localize to the X chromosome [34]. Its restricted distribution in normal tissue but expression in myeloma [25] and in some solid tumours including ovarian cancer [21] and small cell lung carcinoma [22] highlighted Sp17 as a potential immunotherapeutic target in these diseases. We previously reported Sp17 to exhibit the broadest mRNA and protein expression profile in a wide range of haematological cell lines as well as protein expression in primary tumour biopsies from DLBCL [27]. This was of particular importance given previous reports of the paucity of CTA expression in B cell lymphomas [35, 36]. The potential of Sp17 as an immunotherapeutic target was further supported by studies of immunogenic Sp17 CTL epitopes in ovarian cancer [37–39] as well as in myeloma patients [24] and in healthy donors [23]. More recently, Mirandola et al. showed Sp17 to be aberrantly expressed in non-small cell lung cancer patients and it was also immunogenic in these patients [22]. The rationale for the current study was to further validate the Sp17 protein as a potential target in haematological malignancies by studying both its expression and presentation of T cell epitopes. Here, we explored the immunogenicity of Sp17 in a cohort of 31 DLBCL patients, T cell responses being used to demonstrate the presentation of distinct Sp17 epitopes. We have previously reported, in the same cohort of patients, the presence of both CTL and CD4 Th responses to another CTA, the PASD1 protein [29, 30], which is also a member of the CT-X group.

Cytotoxic T cells recognizing Sp17 peptides were detected in 13/31 (41%) of HLA-A^*∗*^0201-positive DLBCL patients after only short-term culture. The magnitude of the CD8 T cell response to Sp17 varied between patients. These results compared favourably with those obtained for the MAGE-A(1–4), LAGE-1, PASD-1, and NY-ESO-1 CTAs in haematological malignancies [13, 29, 40] and for CEA, HER-2/neu, and MAGE-A3 in breast cancer [41]. Our data provide the first experimental validation that Sp17 epitopes are presented and recognized by a T cell response in patients with B cell lymphoma. Our demonstration of a *γ*-IFN response to Sp17 peptides by PBMCs from responder patients after only short-term culture also suggests that Sp17 peptide-specific CTL precursors were present in these individuals. Spontaneous immunity to CTAs, including MAGE-A3 and NY-ESO-1, has also been reported in multiple myeloma [13, 40]. Although outside the scope of the current study investigating epitope presentation, Sp17 might also represent a potential vaccine candidate in DLBCL. To address this possibility, future studies to characterise the functional activity of these T cell populations (e.g., their cytolytic potential) would be needed.

Correlations have been reported between antibody responses to CTAs and prognosis in myeloma [40]. In the present study, a *γ*-IFN response to Sp17 peptides was detected in seven patients with good prognosis GCB-derived DLBCL in addition to the eight patients with poor-prognosis DLBCL (five patients with NGC-derived DLBCL and three patients with transformed DLBCL). These results suggest that Sp17 may be applicable as a therapeutic target regardless of DLBCL subtype. However, given the relatively small number of cases studied here, further study is required to draw any firm conclusions.

Our analysis of sequential blood samples from two DLBCL patients demonstrated a persistent CTL response to Sp17 peptides a year after diagnosis. Both patients remained in remission by the end of this study. Sustained CTL responses to TAAs have been previously reported in myeloma [13] and in anaplastic large cell lymphoma [33, 42]. The persistence of these T cell responses suggests the presence of memory T cells, which might be involved in protective immunity and which also represent potential populations of T cells that could be further stimulated following vaccination [43]. The presence of a significant *γ*-IFN response in these patients both at the time of diagnosis and after one year in remission also suggests the presence of a pool of memory CTL subsets. Such cells could play an important role not only in protective tumour immunity but also in the maintenance of memory CTL responses [44, 45].

A *γ*-IFN response to the Sp17(1) and Sp17(2) peptides was only detected in those patients who were HLA-A^*∗*^0201-positive (except for patient 37). These results, combined with the abrogation of the *γ*-IFN response through depletion of CD8-positive cells or the addition of an anti-MHC class I reagent to CTL lines, provide further evidence for an MHC class I-dependent Sp17 CTL response.

Our study showed that while the Sp17(5) and Sp17(6) peptides were not immunogenic, Sp17(4) induced CD4 Th responses in 4/8 patients expressing Sp17. This is interesting since the responses were not restricted by the different HLA class II allele. The presence of such an epitope recognizable in the context of a variety of different MHC class II molecules could expand the population of patients for whom the peptide could be immunogenic beyond that determined by their MHC class I allele. Moreover, our study shows that CTL as well as CD4 Th responses to Sp17 were recorded in seven patients. A number of studies have demonstrated the potential of using peptide epitopes binding to both MHC class I and class II to achieve optimal immune responses on vaccination [46].

Sp17 represents a potential immunotherapeutic target and is therefore important to correlate the presence of CTL and CD4 *γ*-IFN responses with Sp17 protein expression in tumours. Immunohistochemical labelling with an anti-Sp17 monoclonal antibody confirmed Sp17 expression in 75% (9/12) of the patients tested who exhibited *γ*-IFN responses to Sp17 peptides. Previous studies [13, 40] were able to confirm NY-ESO-1 and MAGE protein expression in those myeloma patients who mounted CTL responses to these CTAs. The low levels and heterogeneity of Sp17 protein expression in the lymphoma cells may explain the absence of detectable Sp17 protein in two patients with a CTL response. Indeed, the biopsy from one patient (patient 5) was a very small bone marrow trephine containing very little lymphoma cells. Furthermore, we previously reported that Sp17 protein expression in DLBCL cell lines was commonly expressed only at low levels that were detectable by Western blotting and not by immunohistochemistry [27]. This may also be the case in primary DLBCL and provides a potential explanation as to why a small number of patients exhibited an anti-Sp17 immune response despite protein expression being undetectable by immunohistochemistry.

It is interesting to note that the majority of the patients with detectable CTL and CD4 Th responses to Sp17 were previously reported to show significant T cell responses to another CTA, the PASD1 protein [29, 30]. The results indicate that both proteins are expressed and immunogenic in this cohort of DLBCL patients. Previous studies have described the presence of more than one CTA antigen in both solid tumours and haematological malignancies such as myeloma and plasmacytoma [47, 48]. Intratumoural variation of CTA expression has been previously described in DLBCL [29], acute myeloid leukaemia [9], and myeloma [49]. The presence of more than one CTA within a tumour, combined with heterogeneity in their protein distribution, thus provides support for the inclusion of multiple CTAs in vaccine development. This approach should maximize the eradication of the tumour cells while minimizing tumour escape variants.

## 5. Conclusions

This study is the first to define immunogenic MHC-presented Sp17 peptides in DLBCL. We showed coordinated CTL and CD4 Th responses to Sp17 in DLBCL patients. The CTL response was MHC class I-restricted and was limited to patients expressing Sp17.

The current results support Sp17 as a potential immunotherapeutic target for patients with Sp17-positive DLBCL or other malignancies expressing this antigen. Since tumours may express more than one CTA, the inclusion of Sp17 in a polyepitope vaccine should increase the chances of successful treatment of patients expressing these antigens.

## Supplementary Material

Supplementary Table 1: Summary of the characterisics of the DLBCL cases investigated here. Patients presented with differing stages and subtype of disease. Their clinical details, treatment protocols and survival status are also listed. Supplementary Table 2: Summary of cytotoxic T lymphocyte (CTL) and CD4 responses to Sp17 and PASD1. Cells from ten patients were able to mount CTL responses to both the Sp17 and PASD1 cancer testis antigens. CD4 Th responses to both the PASD1 and Sp17 antigens were also detected in five of these patients.

## Figures and Tables

**Table 1:** Summary of the CD8 T cell responses to the Sp17 peptides by DLBCL patients.

Patients	Diagnosis	HLA status	Pattern of Sp17 staining	*γ*-IFN response to peptides per 50,000 cells					
				Sp17(1)	Sp17(2)	Sp17(3)	No peptide	HIV-1	PHA
*Significant response*									
1	DLBCL(dn)	A^*∗*^0201+	Cytoplasm and <10% nuclei	84 ± 12	46 ± 10	24 ± 6	12 ± 2	18 ± 4	126 ± 28
2	DLBCL(dn)	A^*∗*^0201+	Scattered nuclei	58 ± 10	74 ± 14	30 ± 8	18 ± 4	20 ± 4	94 ± 16
5	DLBCL(dn)	A^*∗*^0201+	—	48 ± 4	34 ± 16	30 ± 8	16 ± 4	20 ± 4	104 ± 18
7	DLBCL(dn)	A^*∗*^0101+	<10% nuclei	16 ± 4	14 ± 2	48 ± 6	12 ± 2	10 ± 2	124 ± 16
8	DLBCL(dn)	A^*∗*^0201+	ND	102 ± 16	24 ± 4	32 ± 8	20 ± 4	16 ± 4	204 ± 24
9	DLBCL(dn)	A^*∗*^0201+	—	86 ± 12	40 ± 8	38 ± 10	12 ± 4	20 ± 4	128 ± 18
12	DLBCL(dn)	A^*∗*^0201+	Weak cytoplasm and < 5% nuclei	98 ± 14	76 ± 14	42 ± 6	22 ± 2	16 ± 4	154 ± 16
14	DLBCL(dn)	A^*∗*^0201+	ND	66 ± 6	44 ± 8	26 ± 6	12 ± 6	14 ± 2	84 ± 14
18	DLBCL(dn)	A^*∗*^0201+	Weak cytoplasm and <5% nuclei	68 ± 12	54 ± 4	30 ± 8	18 ± 4	20 ± 2	94 ± 16
19	DLBCL(t)	A^*∗*^0201+	<10% nuclei	56 ± 10	62 ± 14	22 ± 4	20 ± 2	18 ± 4	86 ± 12
21	DLBCL(dn)	A^*∗*^0201+	Cytoplasm + scattered < 5% nuclei	46 ± 4	14 ± 2	30 ± 8	12 ± 0	16 ± 2	110 ± 12
22	DLBCL(t)	A^*∗*^0201+	ND	86 ± 8	64 ± 14	40 ± 8	20 ± 4	10 ± 1	88 ± 10
37	DLBCL(dn)	A^*∗*^0101+	<5% nuclei	38 ± 4	24 ± 14	32 ± 4****	12 ± 2	6 ± 1	178 ± 8
39	T cell rich	A^*∗*^0201+	—	28 ± 2	12 ± 2	22 ± 4	10 ± 2	6 ± 1	108 ± 10
*No significant response*									
3	DLBCL(dn)	A^*∗*^0201+	—	28 ± 8	42 ± 6	28 ± 4	16 ± 8	20 ± 4	88 ± 22
4	DLBCL(dn)	A^*∗*^0201+	—	40 ± 6	38 ± 12	22 ± 4	20 ± 4	16 ± 6	132 ± 18
6	DLBCL(dn)	A^*∗*^0201+	—	10 ± 2	12 ± 4	20 ± 2	12 ± 6	6 ± 2	148 ± 10
10	DLBCL(dn)	A^*∗*^0101+	ND	24 ± 4	26 ± 2	16 ± 2	18 ± 2	12 ± 6	106 ± 18
11	DLBCL(dn)	A^*∗*^0201+	ND	36 ± 10	22 ± 4	20 ± 8	18 ± 6	22 ± 4	112 ± 10
13	DLBCL(dn)	A^*∗*^0201+	—	32 ± 8	24 ± 4	18 ± 8	12 ± 6	16 ± 4	98 ± 12
15	DLBCL(dn)	A^*∗*^0101+	ND	16 ± 2	20 ± 6	28 ± 8	10 ± 6	6 ± 2	86 ± 10
16	DLBCL(dn)	A^*∗*^0201+	—	36 ± 6	32 ± 8	30 ± 8	12 ± 6	20 ± 10	94 ± 16
17	DLBCL(dn)	A^*∗*^0201+	ND	16 ± 1	14 ± 2	22 ± 2	16 ± 2	10 ± 4	114 ± 10
20	DLBCL(dn)	A^*∗*^0101+	Cytoplasm + scattered < 5% nuclei	32 ± 1	28 ± 8	36 ± 8	18 ± 4	16 ± 1	134 ± 16
38	DLBCL(dn)	A^*∗*^0201+	ND	26 ± 2	24 ± 4	32 ± 2	20 ± 6	16 ± 10	114 ± 18
40	DLBCL(dn)	A^*∗*^0201-negative, A^*∗*^0101-negative	ND	16 ± 4	14 ± 4	26 ± 4	14 ± 2	10 ± 2	64 ± 12
41	DLBCL(dn)	A^*∗*^0201-negative, A^*∗*^0101-negative	—	18 ± 6	20 ± 4	12 ± 4	10 ± 2	8 ± 2	66 ± 10
42	DLBCL(dn)	A^*∗*^0201-negative, A^*∗*^0101-negative	ND	26 ± 4	22 ± 6	18 ± 6	22 ± 4	20 ± 2	102 ± 16
43	DLBCL(dn)	A^*∗*^0201-negative, A^*∗*^0101-negative	—	24 ± 10	34 ± 14	20 ± 8	22 ± 4	16 ± 2	>500
48	DLBCL(dn)	A^*∗*^0201-negative, A^*∗*^0101-negative	—	16 ± 4	24 ± 14	28 ± 8	22 ± 6	20 ± 2	94 ± 16
49	DLBCL(dn)	A^*∗*^0201-negative, A^*∗*^0101-negative	ND	24 ± 2	18 ± 4	22 ± 6	14 ± 2	10 ± 4	144 ± 18
*Healthy donors*									
1		A^*∗*^0201+	ND	16 ± 4	12 ± 4	16 ± 4	18 ± 4	8 ± 2	68 ± 12
2		A^*∗*^0201+	ND	18 ± 2	20 ± 2	12 ± 4	8 ± 4	6 ± 2	76 ± 10
3		A^*∗*^0201+	ND	32 ± 4	36 ± 4	24 ± 6	16 ± 4	20 ± 2	112 ± 18
4		A^*∗*^0301+	ND	26 ± 10	28 ± 8	12 ± 2	10 ± 4	16 ± 2	88 ± 14

DLBCL(dn): de novo diffuse large B cell lymphoma; DLBCL(t): diffuse large B cell lymphoma transformed; TCR: T cell rich B cell lymphoma. The results +/− are from triplicate ELISPOT cultures. The SD was calculated using standard techniques. Significant *γ*-IFN responses are highlighted in bold. ND, not determined.

**Table 2:** Summary of the CD4 T cell responses to the Sp17 peptides by DLBCL patients.

Patients	MHC class II status	Sp17 protein	*γ*-IFN response to peptides per 50,000 cells					
			Sp17(4)	Sp17(5)	Sp17(6)	No peptide	HIV-1	PHA
*Significant response*								
1	DRB1^*∗*^0102,^*∗*^1104	+	42 ± 4	16 ± 2	14 ± 4	12 ± 2	10 ± 4	92 ± 4
2	DRB1^*∗*^0101,^*∗*^0701	+	28 ± 2	8 ± 2	18 ± 2	10 ± 2	6 ± 1	72 ± 12
5	DRB1^*∗*^0301,^*∗*^0401	−	26 ± 4	12 ± 2	14 ± 2	8 ± 2	4 ± 0	108 ± 10
8	DRB1^*∗*^1501,^*∗*^0803	ND	52 ± 4	12 ± 2	14 ± 4	10 ± 2	6 ± 2	78 ± 14
10	DRB1^*∗*^0101	ND	36 ± 4	20 ± 2	10 ± 4	8 ± 2	4 ± 2	86 ± 6
12	DRB1^*∗*^0401,^*∗*^1401	+	50 ± 2	22 ± 4	28 ± 4	16 ± 2	12 ± 4	128 ± 16
14	DRB1^*∗*^1403,^*∗*^0401	ND	38 ± 4	14 ± 2	24 ± 6	8 ± 2	4 ± 2	78 ± 14
18	DRB1^*∗*^0401,^*∗*^0401	+	32 ± 6	18 ± 2	16 ± 4	6 ± 2	10 ± 2	66 ± 12
*No significant response*								
7	DRB1^*∗*^0301,^*∗*^0701	+	18 ± 6	22 ± 2	26 ± 4	14 ± 2	12 ± 2	58 ± 8
9	DRB1^*∗*^0301,^*∗*^1601	−	16 ± 8	32 ± 4	20 ± 4	18 ± 2	14 ± 4	108 ± 10
15	DRB1^*∗*^1301,^*∗*^1101	ND	10 ± 2	12 ± 2	20 ± 4	10 ± 2	14 ± 2	58 ± 8
19	DRB1^*∗*^0401,^*∗*^0901	+	14 ± 2	18 ± 2	6 ± 1	12 ± 2	8 ± 2	50 ± 8
20	DRB1^*∗*^1104,^*∗*^1501	+	24 ± 4	26 ± 2	14 ± 4	14 ± 2	12 ± 2	72 ± 10
21	DRB1^*∗*^0301,^*∗*^1303	+	10 ± 2	14 ± 2	18 ± 2	9 ± 2	8 ± 2	86 ± 6
39	DRB1^*∗*^0701	−	32 ± 4	26 ± 2	14 ± 2	16 ± 2	14 ± 2	112 ± 16
43	DRB1^*∗*^0101,^*∗*^1101	−	24 ± 4	32 ± 2	28 ± 2	18 ± 4	20 ± 2	98 ± 12
48	DRB1^*∗*^0101,^*∗*^0405	−	28 ± 4	10 ± 2	18 ± 2	18 ± 4	14 ± 2	98 ± 8

DLBCL(dn): de novo diffuse large B cell lymphoma; DLBCL(t): diffuse large B cell lymphoma transformed; TCR: T cell rich B cell lymphoma. The results +/− are from triplicate ELISPOT cultures. The SD was calculated using standard techniques. Significant *γ*-IFN responses are highlighted in bold. ND, not determined.

## References

[1] Stein H., Chan J., Warnke R. Diffuse large B-cell lymphoma, not otherwise specified, WHO Classification of tumours of haematopoietic and lymphoid tissues.

[2] Coiffier B. (2007). Rituximab therapy in malignant lymphoma. *Oncogene*.

[3] Ansell S. M., Lesokhin A. M., Borrello I. (2015). PD-1 blockade with nivolumab in relapsed or refractory Hodgkin's lymphoma. *The New England Journal of Medicine*.

[4] Garfall A. L., Maus M. V., Hwang W.-T. (2015). Chimeric antigen receptor T cells against CD19 for multiple myeloma. *The New England Journal of Medicine*.

[5] Atanackovic D., Arfsten J., Cao Y. (2007). Cancer-testis antigens are commonly expressed in multiple myeloma and induce systemic immunity following allogeneic stem cell transplantation. *Blood*.

[6] Porter D. L., Antin J. H. (2006). Donor leukocyte infusions in myeloid malignancies: new strategies. *Best Practice & Research Clinical Haematology*.

[7] Inaoka R. J., Jungbluth A. A., Gnjatic S. (2012). Cancer/testis antigens expression and autologous serological response in a set of Brazilian non-Hodgkin's lymphoma patients. *Cancer Immunology, Immunotherapy*.

[8] Inaoka R. J., Jungbluth A. A., Baiocchi O. C. G. (2011). An overview of cancer/testis antigens expression in classical Hodgkin's lymphoma (cHL) identifies MAGE-A family and MAGE-C1 as the most frequently expressed antigens in a set of Brazilian cHL patients. *BMC Cancer*.

[9] Atanackovic D., Luetkens T., Kloth B. (2011). Cancer-testis antigen expression and its epigenetic modulation in acute myeloid leukemia. *American Journal of Hematology*.

[10] Srivastava P., Paluch B. E., Matsuzaki J. (2014). Immunomodulatory action of SGI-110, a hypomethylating agent, in acute myeloid leukemia cells and xenografts. *Leukemia Research*.

[11] Pellat-Deceunynck C., Mellerin M.-P., Labarrière N. (2000). The cancer germ-line genes MAGE-1, MAGE-3 and PRAME are commonly expressed by human myeloma cells. *European Journal of Immunology*.

[12] Lim S. H., Wang Z., Chiriva-Internati M., Xue Y. (2001). Sperm protein 17 is a novel cancer-testis antigen in multiple myeloma. *Blood*.

[13] Goodyear O., Piper K., Khan N. (2005). CD8+T cells specific for cancer germline gene antigens are found in many patients with multiple myeloma, and their frequency correlates with disease burden. *Blood*.

[14] De Carvalho F., Alves V. L. F., Braga W. M. T., Xavier C. V., Colleoni G. W. B. (2013). MAGE-C1/CT7 and MAGE-C2/CT10 are frequently expressed in multiple myeloma and can be explored in combined immunotherapy for this malignancy. *Cancer Immunology, Immunotherapy*.

[15] Taylor B. J., Reiman T., Pittman J. A. (2005). SSX cancer testis antigens are expressed in most multiple myeloma patients: Co-expression of SSX1, 2, 4, and 5 correlates with adverse prognosis and high frequencies of SSX-positive PCs. *Journal of Immunotherapy*.

[16] Gjerstorff M. F., Andersen M. H., Ditzel H. J. (2015). Oncogenic cancer/testis antigens: prime candidates for immunotherapy. *Oncotarget *.

[17] Caballero O. L., Chen Y.-T. (2009). Cancer/testis (CT) antigens: Potential targets for immunotherapy. *Cancer Science*.

[18] Seledtsov V. I., Goncharov A. G., Seledtsova G. V. (2015). Clinically feasible approaches to potentiating cancer cell-based immunotherapies. *Human Vaccines & Immunotherapeutics*.

[19] Xiang S. D., Gao Q., Wilson K. L., Heyerick A., Plebanski M. (2015). Mapping T and B cell epitopes in sperm protein 17 to support the development of an ovarian cancer vaccine. *Vaccine*.

[20] Song J.-X., Cao W.-L., Li F.-Q., Shi L.-N., Jia X. (2012). Anti-Sp17 monoclonal antibody with antibody-dependent cell-mediated cytotoxicity and complement-dependent cytotoxicity activities against human ovarian cancer cells. *Medical Oncology*.

[21] Straughn J. M., Shaw D. R., Guerrero A. (2004). Expression of sperm protein 17 (Sp17) in ovarian cancer. *International Journal of Cancer*.

[22] Mirandola L., Figueroa J. A., Phan T. T. (2015). Novel antigens in non-small cell lung cancer: SP17, AKAP4, and PTTG1 are potential immunotherapeutic targets. *Oncotarget *.

[23] Chiriva-Internati M., Wang Z., Salati E., Wroblewski D., Lim S. H. (2002). Successful generation of sperm protein 17 (Sp17)-specific cytotoxic T lymphocytes from normal donors: Implication for tumour-specific adoptive immunotherapy following allogeneic stem cell transplantation for Sp17-positive multiple myeloma. *Scandinavian Journal of Immunology*.

[24] Chiriva-Internati M., Wang Z., Pochopien S., Salati E., Lim S. H. (2003). Identification of a sperm protein 17 CTL epitope restricted by HLA-A1. *International Journal of Cancer*.

[25] Chiriva-Internati M., Wang Z., Xue Y., Bumm K., Hahn A. B., Lim S. H. (2001). Sperm protein 17 (Sp17) in multiple myeloma: Opportunity for myeloma-specific donor T cell infusion to enhance graft-versus-myeloma effect without increasing graft-versus-host disease risk. *European Journal of Immunology*.

[26] Lacy H. M., Sanderson R. D. (2001). Sperm protein 17 is expressed on normal and malignant lymphocytes and promotes heparan sulfate-mediated cell-cell adhesion. *Blood*.

[27] Liggins A. P., Lim S. H., Soilleux E. J., Pulford K., Banham A. H. (2010). A panel of cancer-testis genes exhibiting broad-spectrum expression in haematological malignancies.. *Cancer immunity : a journal of the Academy of Cancer Immunology*.

[28] Arnaboldi F., Menon A., Menegola E. (2014). Sperm Protein17 is an oncofetal antigen: A lesson from a murine model. *International Reviews of Immunology*.

[29] Ait-Tahar K., Liggins A. P., Collins G. P. (2009). Cytolytic T-cell response to the PASD1 cancer testis antigen in patients with diffuse large B-cell lymphoma. *British Journal of Haematology*.

[30] Ait-Tahar K., Liggins A. P., Collins G. P. (2011). CD4-positive T-helper cell responses to the PASD1 protein in patients with diffuse large B-cell lymphoma. *Haematologica*.

[31] Bunce M., O'Neill C. M., Barnardo M. C. N. M. (1995). Phototyping: comprehensive DNA typing for HLA‐A, B, C, DRB1, DRB3, DRB4, DRB5 & DQB1 by PCR with 144 primer mixes utilizing sequence‐specific primers (PCR‐SSP). *Tissue Antigens*.

[32] Hans C. P., Weisenburger D. D., Greiner T. C. (2004). Confirmation of the molecular classification of diffuse large B-cell lymphoma by immunohistochemistry using a tissue microarray. *Blood*.

[33] Ait-Tahar K., Cerundolo V., Banham A. H. (2006). B and CTL responses to the ALK protein in patients with ALK-positive ALCL. *International Journal of Cancer*.

[34] Grizzi F., Mirandola L., Qehajaj D., Cobos E., Figueroa J. A., Chiriva-Internati M. (2015). Cancer-testis antigens and immunotherapy in the light of cancer complexity. *International Reviews of Immunology*.

[35] Huang S., Preuss K.-D., Xie X., Regitz E., Pfreundschuh M. (2002). Analysis of the antibody repertoire of lymphoma patients. *Cancer Immunology, Immunotherapy*.

[36] Xie X., Wacker H. H., Huang S. (2003). Differential expression of cancer testis genes in histological subtypes of non-Hodgkin's lymphomas. *Clinical Cancer Research*.

[37] Chiriva-Internati M., Weidanz J. A., Yu Y. (2008). Sperm protein 17 is a suitable target for adoptive T-cell-based immunotherapy in human ovarian cancer. *Journal of Immunotherapy*.

[38] Chiriva-Internati M., Yu Y., Mirandola L. (2010). Cancer testis antigen vaccination affords long-term protection in a murine model of ovarian cancer. *PLoS ONE*.

[39] Xiang S. D., Gao Q., Wilson K. L., Heyerick A., Plebanski M. (2015). A nanoparticle based Sp17 peptide vaccine exposes new immuno-dominant and species cross-reactive B cell epitopes. *Vaccines*.

[40] Van Rhee F., Szmania S. M., Zhan F. (2005). NY-ESO-1 is highly expressed in poor-prognosis multiple myeloma and induces spontaneous humoral and cellular immune responses. *Blood*.

[41] Inokuma M., Dela Rosa C., Schmitt C. (2007). Functional T cell responses to tumor antigens in breast cancer patients have a distinct phenotype and cytokine signature. *The Journal of Immunology*.

[42] Passoni L., Scardino A., Bertazzoli C. (2002). ALK as a novel lymphoma-associated tumor antigen: Identification of 2 HLA-A2.1-restricted CD8+ T-cell epitopes. *Blood*.

[43] Baumgaertner P., Rufer N., Devevre E. (2006). Ex vivo detectable human CD8 T-cell responses to cancer-testis antigens. *Cancer Research*.

[44] Provine N. M., Larocca R. A., Aid M. (2016). Immediate dysfunction of vaccine-elicited CD8+ T cells primed in the absence of CD4+ T cells. *The Journal of Immunology*.

[45] Ostrand-Rosenberg S. (2005). CD4+ T lymphocytes: A critical component of antitumor immunity. *Cancer Investigation*.

[46] Zeng G. (2001). MHC class II-restricted tumor antigens recognized by CD4+ T cells: New strategies for cancer vaccine design. *Journal of Immunotherapy*.

[47] Atanackovic D., Blum I., Cao Y. (2006). Expression of cancer-testis antigens as possible targets for antigen-specific immunotherapy in head and neck squamous cell carcinoma. *Cancer Biology & Therapy*.

[48] Condomines M., Hose D., Raynaud P. (2007). Cancer/testis genes in multiple myeloma: Expression patterns and prognosis value determined by microarray analysis. *The Journal of Immunology*.

[49] Dhodapkar M. V., Osman K., Teruya-Feldstein J. (2003). Expression of cancer/testis (CT) antigens MAGE-A1, MAGE-A3, MAGE-A4, CT-7, and NY-ESO-1 in malignant gammopathies is heterogeneous and correlates with site, stage and risk status of disease.. *Cancer immunity : a journal of the Academy of Cancer Immunology*.

