# Supplementary material for: Sp17 Protein Expression and Major Histocompatibility Class I and II Epitope Presentation in Diffuse Large B Cell Lymphoma Patients
Source: Adv Hematol. 2017 Oct 24;2017:6527306. doi: 10.1155/2017/6527306 (PMC5674480; doi:10.1155/2017/6527306)
Supplement: Supplementary file 1 — Supplementary Table 1: Summary of the characterisics of the DLBCL cases investigated here. Patients presented with differing stages and subtype of disease. Their clinical details, treatment protocols and survival status are also listed. Supplementary Table 2: Summary of cytotoxic T lymphocyte (CTL) and CD4 responses to Sp17 and PASD1. Cells from ten patients were able to mount CTL responses to both the Sp17 and PASD1 cancer testis antigens. CD4 Th responses to both the PASD1 and Sp17 antigens were also detected in five of these patients. [file 6527306.f1.docx]

**Supplementary Table 1. Summary of the characteristics of the DLBCL cases studied here**. The cohort of patients was previously studied in two earlier studies*,** on the immunogenicity of the PASD1 cancer testis antigen. Patients presented with differing stages and subtype of disease. Their clinical details, treatment protocols and survival status are also listed.

| **Patients** | **Diagnosis** | **Subtype**^#^ | **Stage** | **IPI** | **Sex** | **Age (years)** | **Treatment** | **Status at the time of study** |
| --- | --- | --- | --- | --- | --- | --- | --- | --- |
| 1 | DLBCL (dn) | NGC | 1 | 1 | F | 23 | CHOP-R | CR (21 months) |
| 2 | DLBCL(dn) | GCB | 3 | 3 | M | 67 | CHOP-R + MTX + RX | CR (20 months) |
| 3 | DLBCL(dn) | GCB | 3 | 3 | M | 81 | VIN/PRED | Died (22 months) |
| 4 | DLBCL(dn) | NGC | 1 | 2 | F | 76 | CHOP-R | PR (29 months) |
| 5 | DLBCL(dn) | GCB | 1 | 0 | M | 52 | CHOP-R + RX | CR (12 months) |
| 6 | DLBCL(dn) | NGC | 2 | 1 | M | 21 | CHOP-R | Died (19 months) |
| 7 | DLBCL(dn) | GCB | 2 | 0 | M | 49 | CHOP-R + MTX | PR (19 months) |
| 8 | DLBCL(dn) | GCB | 1 | 0 | M | 63 | CHOP-R | CRU (24 months) |
| 9 | DLBCL(dn) | NGC | 3 | 2 | F | 71 | CHOP-R | PR (23 months) |
| 10 | DLBCL(dn) | GCB | 1 | 0 | F | 60 | CHOP-R + RICE + ESHAP+ BEAM+ TX | CR (13 months) |
| 11 | DLBCL(dn) | GCB | 1 | 1 | M | 38 | CODOX-M + RX | PR (17 months) |
| 12 | DLBCL(dn) | GCB | 1 | 0 | F | 59 | CHOP-R | CR (22 months) |
| 13 | DLBCL(dn) | GCB | 3 | 3 | M | 67 | CHOP-R + MTX | PR (17 months) |
| 14 | DLBCL(dn) | NGC | 3 | 2 | M | 63 | CHOP-R + MTX | CR (12 months relapse 2 months) |
| 15 | DLBCL(dn) | NGC | 3 | 3 | M | 85 | VIN/PRED | Died (6 months) |
| 16 | DLBCL(dn) | GCB | 2 | 2 | M | 59 | CHOP-R | CR (22 months) |
| 17 | DLBCL(dn) | GCB | 3 | 4 | M | 60 | CHOP-R | CR (17 months) |
| 18 | DLBCL(dn) | NGC | 4 | 4 | M | 74 | CHOP-R | CR (14 months) |
| 19 | DLBCL(dn) | GCB | 4 | 2 | M | 56 | CHOP-R + RX | Died (19 months) |
| 20 | DLBCL(dn) | NGC | 2 | 2 | F | 70 | CHOP-R | Died (2 months) |
| 21 | DLBCL(dn) | GCB | 1 | 3 | M | 73 | CHOP-R | PR (23 months) |
| 22 | DLBCL(dn) | GCB | 3 | 1 | M | 53 | CHOP-R | PR (24 months) |
| 37 | DLBCL(t) | ND | 1 | 2 | M | 59 | CHOP-R + RX | CR (22 months) |
| 38 | DLBCL(t) | ND | 3 | 2/3 | M | 71 | PMitCEBO + PRED + RX + VIN | CR (12 months) |
| 39 | DLBCL(t) | ND | 4 | 2 | F | 39 | CHOP-R | Died (6 months) |
| 40 | DLBCL(t) | ND | 3 | 3 | M | 64 | CHOP-R | Died (4 months) |
| 41 | DLBCL(t) | ND | 4 | 4 | F | 60 | CHOP-R + CNOP-R | CR (29 months) |
| 42 | DLBCL(t) | ND | 2 | 1 | F | 54 | CHOP-R | PR (5 months) |
| 43 | DLBCL(t) | ND | 4 | 2 | F | 60 | CHOP-R + RX | CR (24 months) |
| 48 | T-cell rich | ND | 2 | 0 | M | 51 | CHOP-R | CR (18 months) |
| 49 | T-cell rich | ND | 3 | 4 | M | 74 | CHOP-R | PR (18 months) |

IPI, International Prognostic Index; DLBCL(dn): *De novo* diffuse large B-cell lymphoma; DLBCL(t): diffuse large B-cell lymphoma transformed;TCR: T-cell rich B-cell lymphoma; # subtyped according to expression of CD10, BCL-6 and MUM1; GCB: germinal center-derived; NGC: non-germinal center-derived; CHOP-R: cyclophosphamide, doxorubicin, vincristine, prednisolone,rituximab; MTX: intrathecal methotrexate; RX: radiotherapy; PRED: prednisolone; VIN: vinblastine; RICE: rituximab, ifosfamide,carboplatin, etoposide; ESHAP: etoposide, methyprednisolone, cytarabine, cisplatin; TX: autologous transplant; CODOX-M: cyclophosphamide, vincristine, doxorubicin,methotrexate; BEAM-BCNU: (bis-chloro-ethyl nitrosourea), etoposide, cytarabine, melphalan; CNOP-R: cyclophosphamide, mitoxantrone, vincristine, prednisolone, rituximab; PMITCEBO-R: prednisolone, mitoxantrone, cyclophosphamide, etoposide, bleomycin, vincristine, rituximab; IVAC: ifosfamide, etoposide, cytarabine; CR: complete response; PR: partial response: CRU: complete remission unconfirmed; ND: Not determined

*K. Ait-Tahar, A.P. Liggins, G.P. Collins, et al., "Cytolytic T-cell response to the PASD1 cancer testis antigen in patients with diffuse large B-cell lymphoma," *British Journal of Haematology*, vol. 146, no. 4, pp. 396-407, 2009.

**Ait-Tahar K, Liggins AP, Collins GP, Campbel A, Barnardo M, Cabes M et al. CD4 Th responses to the PASD1 protein in patients with diffuse large B-cell lymphoma. *Haematologica,* 2011; 96(1):78-86.

**Supplementary Table 2. Summary of cytotoxic T lymphocyte (CTL) and CD4 responses to Sp17 and PASD1.** The T-cell responses of patients to the Sp17 antigen presented in the current study were compared to their T-cell responses to the PASD1 cancer testis antigen (CTA) described previously. Cells from ten patients were able to mount CTL responses to both Sp17 and PASD1 CTAs. CD4 Th responses to both the PASD1 and Sp17 antigens were also detected in five of these patients.

| **Patient** | **Diagnosis** | **Subtype*≠** | **Sp17 CTL response** | **Sp17 CD4 response** | **PASD1 CTL response*** | **PASD1 CD4**  **response**** |
| --- | --- | --- | --- | --- | --- | --- |
| 1 | DLBCL (dn) | NGC | + | + | + | + |
| 2 | DLBCL(dn) | GCB | + | + | + | + |
| 3 | DLBCL(dn) | GCB | - | ND | + | + |
| 4 | DLBCL(dn) | NGC | - | ND | + | + |
| 5 | DLBCL(dn) | GCB | + | + | + | ND |
| 6 | DLBCL(dn) | NGC | - | ND | + | ND |
| 7 | DLBCL(dn) | GCB | + | - | + | ND |
| 8 | DLBCL(dn) | GCB | + | + | + | + |
| 9 | DLBCL(dn) | NGC | + | - | + | - |
| 10 | DLBCL(dn) | GCB | - | + | + | - |
| 11 | DLBCL(dn) | GCB | - | ND | + | - |
| 12 | DLBCL(dn) | GCB | + | + | + | + |
| 13 | DLBCL(dn) | GCB | - | ND | + | - |
| 14 | DLBCL(dn) | NGC | + | + | + | + |
| 15 | DLBCL(dn) | NGC | - | - | + | - |
| 16 | DLBCL(dn) | GCB | - | ND | + | ND |
| 17 | DLBCL(dn) | GCB | - | ND | + | - |
| 18 | DLBCL(dn) | NGC | + | + | + | - |
| 19 | DLBCL(dn) | GCB | + | - | - | - |
| 20 | DLBCL(dn) | NGC | - | - | - | - |
| 21 | DLBCL(dn) | GCB | + | - | - | - |
| 22 | DLBCL(dn) | GCB | + | ND | - | ND |
| 37 | DLBCL(t) | ND | + | ND | + | - |
| 38 | DLBCL(t) | ND | - | ND | + | ND |
| 39 | DLBCL(t) | ND | + | - | - | - |
| 40 | DLBCL(t) | ND | - | ND | ND | - |
| 41 | DLBCL(t) | ND | - | ND | ND | + |
| 42 | DLBCL(t) | ND | - | ND | ND | ND |
| 43 | DLBCL(t) | ND | - | - | ND | + |
| 48 | T-cell rich | ND | - | - | ND | ND |
| 49 | T-cell rich | ND | - | ND | - | ND |

DLBCL(dn): *De novo* diffuse large B cell lymphoma; DLBCL(t): diffuse large B cell lymphoma transformed; TCR: T-cell rich B-cell lymphoma; ≠ subtyped acccording to expression of CD10, BCL-6,and MUM-1; GCB: germinal center-derived; NGC: non-germinal center-derived. ND: Not Done. The results +/- are from triplicate ELISPOT

cultures. The SD was calculated using standard techniques. Significant γ-IFN responses are highlighted. ND, not determined. Green shading shows T-cell responses to Sp17 and PASD1 while yellow shading denotes a γ-IFN responses to either Sp17 or PASD1

*[Ait-Tahar K](http://www.ncbi.nlm.nih.gov/pubmed/?term=Ait-Tahar%20K%5BAuthor%5D&cauthor=true&cauthor_uid=19552722), [Liggins AP](http://www.ncbi.nlm.nih.gov/pubmed/?term=Liggins%20AP%5BAuthor%5D&cauthor=true&cauthor_uid=19552722), [Collins GP](http://www.ncbi.nlm.nih.gov/pubmed/?term=Collins%20GP%5BAuthor%5D&cauthor=true&cauthor_uid=19552722), [Campbell A](http://www.ncbi.nlm.nih.gov/pubmed/?term=Campbell%20A%5BAuthor%5D&cauthor=true&cauthor_uid=19552722), [Barnardo M](http://www.ncbi.nlm.nih.gov/pubmed/?term=Barnardo%20M%5BAuthor%5D&cauthor=true&cauthor_uid=19552722), [Lawrie C](http://www.ncbi.nlm.nih.gov/pubmed/?term=Lawrie%20C%5BAuthor%5D&cauthor=true&cauthor_uid=19552722) et al. Cytolytic T-cell response to the PASD1 cancer testis antigen in patients with diffuse large B-cell lymphoma. [*Br J Haematol.*](http://www.ncbi.nlm.nih.gov/pubmed/19552722) 2009; 146(4):396-407.

**Ait-Tahar K, Liggins AP, Collins GP, Campbel A, Barnardo M, Cabes M et al. CD4 Th responses to the PASD1 protein in patients with diffuse large B-cell lymphoma. *Haematologica,* 2011; 96(1):78-86.
